# Positioning SUMO as an immunological facilitator of oncolytic viruses for high-grade glioma

**DOI:** 10.3389/fcell.2023.1271575

**Published:** 2023-10-04

**Authors:** Paramesh V. Karandikar, Lyle Suh, Jakob V. E. Gerstl, Sarah E. Blitz, Qing Rui Qu, Sae-Yeon Won, Florian A. Gessler, Omar Arnaout, Timothy R. Smith, Pier Paolo Peruzzi, Wei Yang, Gregory K. Friedman, Joshua D. Bernstock

**Affiliations:** ^1^ T. H. Chan School of Medicine, University of Massachusetts, Worcester, MA, United States; ^2^ Department of Neurosurgery, Brigham and Women’s Hospital, Harvard Medical School, Boston, MA, United States; ^3^ Department of Neurosurgery, University of Rostock, Rostock, Germany; ^4^ Department of Anesthesiology, Multidisciplinary Brain Protection Program, Duke University Medical Center, Durham, NC, United States; ^5^ Department of Neuro-Oncology, Division of Cancer Medicine, MD Anderson Cancer Center, Houston, TX, United States; ^6^ David H. Koch Institute for Integrative Cancer Research, Massachusetts Institute of Technology, Cambridge, MA, United States

**Keywords:** SUMO, SUMOtherapeutics, oncolytic viruses, cancer immunotherapies, high-grade glioma

## Abstract

Oncolytic viral (OV) therapies are promising novel treatment modalities for cancers refractory to conventional treatment, such as glioblastoma, within the central nervous system (CNS). Although OVs have received regulatory approval for use in the CNS, efficacy is hampered by obstacles related to delivery, under-/over-active immune responses, and the “immune-cold” nature of most CNS malignancies. SUMO, the Small Ubiquitin-like Modifier, is a family of proteins that serve as a high-level regulator of a large variety of key physiologic processes including the host immune response. The SUMO pathway has also been implicated in the pathogenesis of both wild-type viruses and CNS malignancies. As such, the intersection of OV biology with the SUMO pathway makes SUMOtherapeutics particularly interesting as adjuvant therapies for the enhancement of OV efficacy alone and in concert with other immunotherapeutic agents. Accordingly, the authors herein provide: 1) an overview of the SUMO pathway and its role in CNS malignancies; 2) describe the current state of CNS-targeted OVs; and 3) describe the interplay between the SUMO pathway and the viral lifecycle and host immune response.

## 1 Introduction

### 1.1 SUMO

SUMO, Small Ubiquitin-Like Modulator, is a family of proteins involved in the high-level regulation of cellular homeostasis and responses to physiologic stressors via post-translational modification. Of the known SUMO paralogs, SUMO-1 and SUMO-2/3 are of the greatest clinical significance (Liang et al., 2016; Sahin et al., 2022). Presently, over 14,000 SUMO binding domains have been found within the human cell (Hendriks et al., 2018). The span of subsequent potential therapeutic application includes: ischemic stroke (Karandikar et al., 2023), cardiovascular and neurodegenerative disease (Lee et al., 2016; Bernstock et al., 2017a; Bernstock et al., 2020a; Chen et al., 2021), and oncology (Seeler and Dejean, 2017). Additionally, the druggable characteristics of the SUMO pathway have made it of particular research interest; for example, high throughput screening has allowed for the identification of drugs enhancing SUMO conjugation via inhibition of either microRNAs 182/183 or SUMO-specific protease 2 (Bernstock et al., 2016; Bernstock et al., 2018).

**FIGURE 1:**
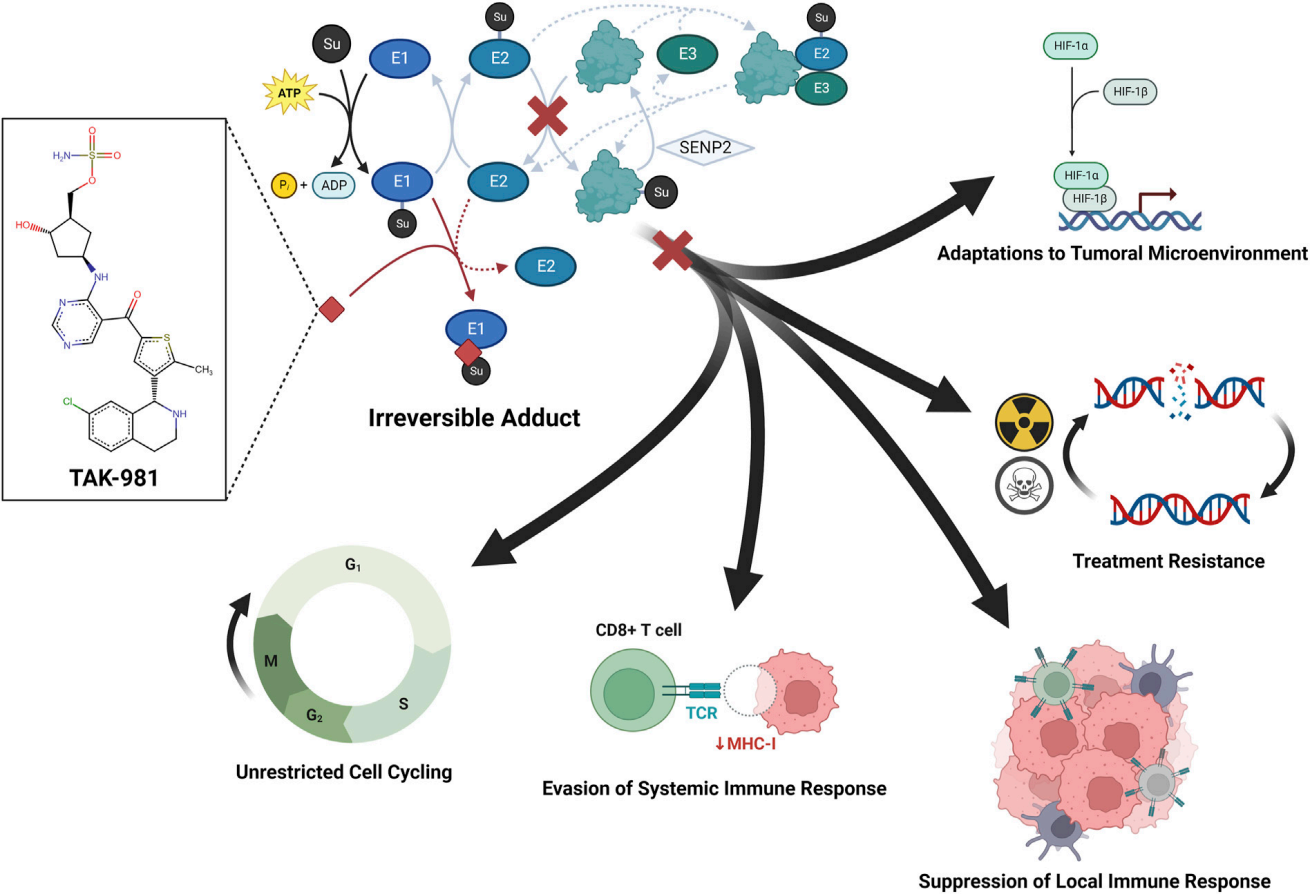
SUMOtherapeutics as a means to counter tumor adaptations.

The high-level regulatory function of SUMO extends to oncogenes in a variety of cancers (Lee et al., 2017). As such, the implication of SUMO in the pathogenesis of resistant cancers has positioned SUMOtherapeutics as potential anti-cancer agents and immunotherapeutic adjuvants (Seeler and Dejean, 2017). The first SUMOtherapeutic, TAK-981 (Takeda Pharmaceuticals, Tokyo, Japan), has been reported to induce cell-cycle arrest (Hanel et al., 2022; Kim et al., 2023), deplete Treg populations (Weitz et al., 2022) and spur immune activation (Khattar et al., 2019; Lightcap et al., 2021; Kumar et al., 2022), giving rise to increasing efforts to apply it to a wide set of cancers (Langston et al., 2021) (Figure 1). TAK-981 is presently being investigated for advanced non-small cell lung cancer, cervical cancer, microsatellite-stable colorectal cancer, refractory or relapsed diffuse large B-cell lymphoma, and follicular lymphoma (NCT03648372). Additionally, TAK-981 is under evaluation as an adjunct for use with the immune checkpoint inhibitor pembrolizumab in advanced solid tumors (NCT04381650) and anti-CD38 monoclonal antibody mezagitamab for multiple myeloma (NCT04776018). Trials with TAK-981 as an adjuvant for anti-CD20 monoclonal antibody rituximab for refractory non-Hodgkin’s lymphoma (NCT04074330) and for cetuximab and avelumab for head and neck cancer (NCT04065555) are complete but are yet to report results. Although no SUMOtherapeutic agents are presently under investigation for CNS malignancies, an increasing body of research is demonstrating the integral relationship between SUMO and CNS cancer.

**FIGURE 2:**
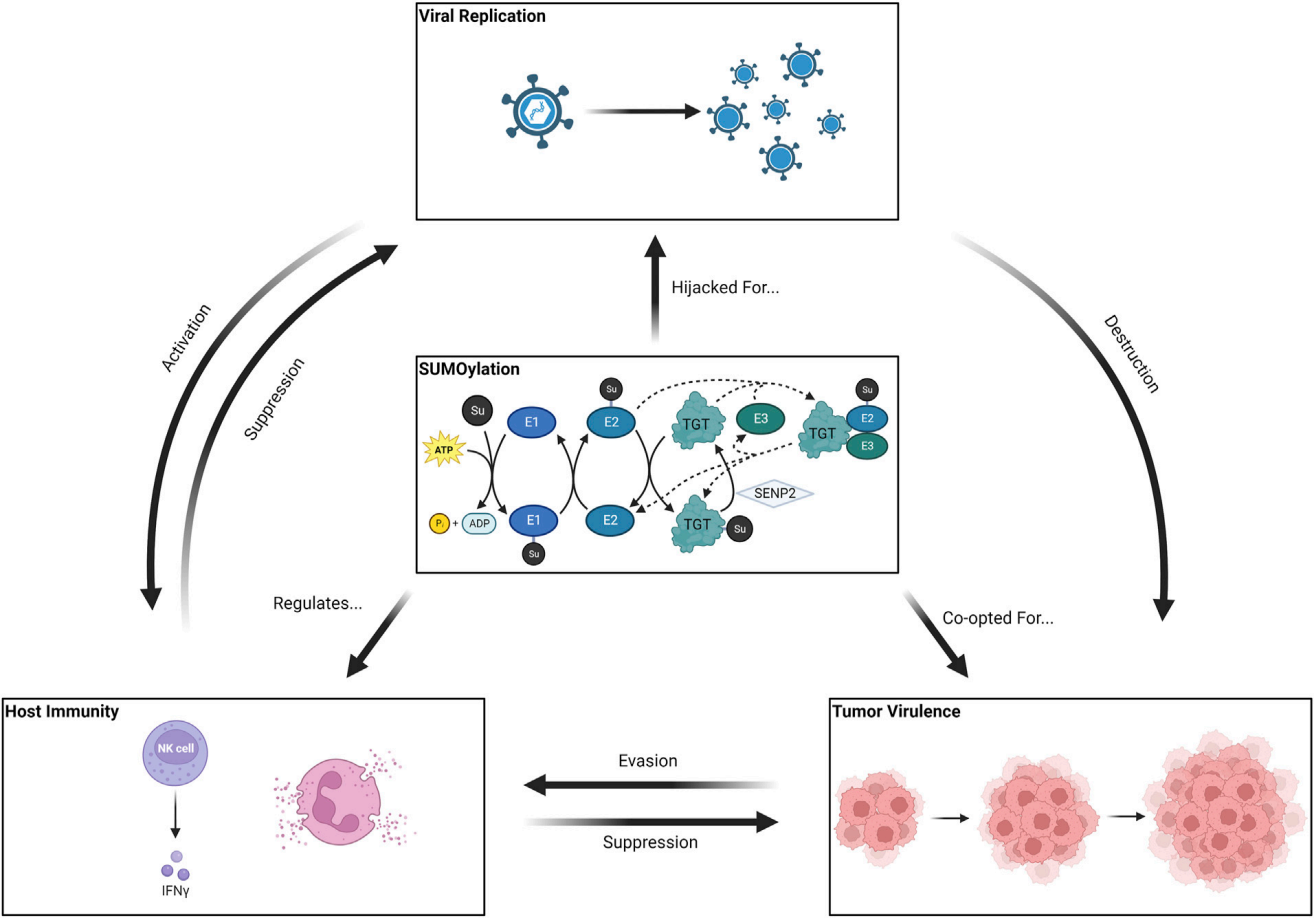
SUMO and its impact on host immunity, viral physiology, and cancer.

The role of the SUMO pathway in glioblastoma (GBM) pathogenesis has been demonstrated in several basic science works. Yang et al. (2013) obtained specimens from 58 patients with astrocytic cerebral malignancies and subjected them to histopathological and biochemical analysis, finding 28-fold increases in SUMO-conjugated protein concentrations in GBM samples as compared to controls. Interestingly, 12- and 17-fold increases in SUMOylated protein concentration were also observed in Grade II and Grade III astrocytomas, respectively (Yang et al., 2013). Subsequent investigation by Bellail et al. (2014); Bellail and Hao (2016) found that SUMO-conjugation of cyclin-dependent kinase 6 (CDK6) via Ubc9 ligase (also known as E2) effectively prevented ubiquitinylation and subsequent degradation, thereby enabling runaway cellular replication (Bellail et al., 2014; Bellail and Hao, 2016). Subsequently, the same authors identified a small molecule inhibitor of SUMOylation that induced ubiquitinylation of SUMO1 and demonstrated its efficacy in retarding the progression of patient-derived xenografts (LN-229, also known as CRL-2611) in mice (Bellail et al., 2021). Furthermore, the SUMO pathway has been shown to be involved with GBM virulence factors such as resistance to double-stranded DNA breaks and the ability to thrive in the hypoxic tumor microenvironment via adaptations such as the Warburg effect and HIF-1α upregulation. Bernstock et al. (2017b) assessed the effect of temozolomide, known to decrease SUMOylation in other cell lines, on the proteome of human GBM lines and observed contradictory increases in SUMO that were not significantly different from the negative controls. Furthermore, upon treating GBM cultures with topotecan, a known GBM chemosensitizer and putative SUMOylation inhibitor, a decrease in CDK6 SUMOylation, HIF-1α expression, and concomitant G1/S transition block were apparent (Bernstock et al., 2017b). Other preclinical work has highlighted the importance of SUMO in GBM virulence by way of CRMP2-mediated proliferation (Wang and Ji, 2019), vimentin-mediated cell motility, and ATR/NUSAP1-mediated chemoresistance (Zhao et al., 2020). As a result, the importance of SUMO in GBM pathogenesis and its putative interface with host immunity and viral physiology makes it uniquely suitable as a therapeutic target (Figure 2).

SUMO has been implicated in adaptations to the hypoxic tumor environment, robust DNA repair capabilities, induction of anergy in infiltrating immune cells, non-stop reproduction, and escape from systemic immune responses. TAK-981 is the first SUMOtherapeutic anti-cancer small molecule and functions as a suicide inhibitor of E1 by forming an irreversible adduct and prevention of engagement of the E2-conjugating enzyme, and subsequent ligation of protein substrates. Preclinical literature has demonstrated anti-cancer properties of TAK-981 by way of immune activation (Khattar et al., 2019; Lightcap et al., 2021; Kumar et al., 2022) and induction of cell-cycle arrest (Hanel et al., 2022; Kim et al., 2023).

*SUMO serves in varying capacities as a regulator, inadvertent facilitator, and hostage of host immunity, viral replication, and tumor virulence. SUMO is necessary for both activation and downregulation of the Type I interferon response, which in turn is critical for mounting an effective defense against both infection and tumor propagation. The role of SUMO in managing the cellular response against physiological stressors also enables SUMO-upregulating tumors to thrive notwithstanding the hypoxic microenvironment or genotoxic insults such as chemotherapy and radiotherapy. Finally, many wild-type viruses and their respective OV-candidate recombinants utilize components or enzymes within the SUMO pathway to evade the host antiviral response and generate greater cytotoxic (or oncolytic) effects.*

### 1.2 Oncolytic viruses in the CNS

The oncolytic properties of viruses were first reported by Dr. George Dock as early as 1902 (Dock, 1904; Kelly and Russell, 2007). More recently, the advent of genetic engineering technologies has enabled oncolytic viruses (OVs) to be a viable clinical tool. A form of immunotherapy, OV therapy is under investigation for cancers associated with poor response to conventional chemo-/radiotherapy and/or surgical resection (Friedman et al., 2018; Bernstock et al., 2020b; Bernstock et al., 2020c; Bernstock et al., 2021; Friedman et al., 2021; Stavrakaki et al., 2021; Bernstock et al., 2023a). Currently, five viruses have received regulatory approval as lone agents or adjuvant/salvage therapies in addition to conventional chemotherapy/radiotherapy regimens. Notably, the oncolytic herpesvirus recombinant G47Δ received approval in Japan for treatment of refractory/recurrent glioma—the first OV to be approved for the indication (Todo et al., 2022a; Shalhout et al., 2023). However, the promising results of extra-CNS OV applications remain to be replicated more broadly in high-grade CNS malignancies. Several characteristics have been suggested to explain these results: the immune privilege created within the tumor, insufficient viral oncotropism, rapid viral clearance, and insufficient/excessive immunogenicity (Bernstock et al., 2019; Monie et al., 2021).

In particular, GBM is known to generate an immune-suppressed environment within the tumor core despite penetration by both CNS-resident microglia and monocytes attracted from the periphery (Hambardzumyan et al., 2016). GBMs have been observed to consist of up to 50% tumor-associated macrophages by mass, with increased abundance correlated with worse prognosis as well as aggressiveness (Sørensen et al., 2018). Similarly, GBMs are also associated with the recruitment of regulatory T-cells and reduced expression of neoepitopes suitable for generating a more acute immune response (Lu-Emerson et al., 2013). Furthermore, TAMs, while anergic against the tumor itself, act against injected OVs by both phagocytosis of virion as well as formation of physical barriers that impede their dissemination (Liu et al., 2023). Of note, Delwar et al. (2018) imputed a STAT1/3 dependent mechanism for microglial inactivation of oHSV-1 strains in a U87 xenograft model. STAT1 has, upon SUMOylation, been reported to preferentially downregulate the Type II Interferon response while maintaining the Type I response, thus preferentially enhancing antiviral immunity (El-As et al., 2020). As such, GBMs have a combination of virulence and immune stealth that enables escape from innate immune defenses and OVs alike (Jackson et al., 2019).

## 2 State of the art in CNS-targeted oncolytic viruses

### 2.1 Viral vectors

To date, several human and nonhuman viruses have been assessed as OV candidates (Fudaba and Wakimoto, 2023). In general, good OV candidates have tropism to the target tissue or tumor cells and are primed to replicate within only malignant cells. They should also generate a sufficient immune response (largely secondary to an innate oncolytic process) and immune activation to destroy the tumor while preventing runaway inflammation and viremia. Although neurotropic species enjoy tropism to cell phenotypes found within the CNS by default, they pose the risk of chronic neuroinflammation if the body fails to clear the virion after a therapeutic interval (Monie et al., 2021). In contrast, non-neurotropic species avoid this risk yet require significant engineering or targeted delivery methods to ensure selective infection (Monie et al., 2021). Another important differentiating factor is the replication competence of the vector in question: wild-type viruses will replicate according to their natural tropism whereas edited viruses can be engineered to replicate conditionally or not at all. While lytic replication was previously believed to be the primary mechanism of OV effect, recent reports of the role of viral-mediated immunogenicity (even with inactivated OVs) in generating anti-tumor immunity have made it a significant subject of inquiry (Davola and Mossman, 2019). Even so, each candidate offers a unique set of advantages and disadvantages (Table 1).

**TABLE 1:** Selected OV Candidates for CNS applications.

	Family	Species	Recombinant/Variant	Advantages	Limitations	Modifications		References
dsDNA	Herpesviridae	HSV-1	HSV1716	‐ Large payload capacity (20–30 Kb) ‐ Genes contributing to pathogenesis are not necessary for replication ‐ Endogenous neurotropism ‐ Endogenous cytolytic activity ‐ Effective antiviral agents available as means of control	- High prevalence of seropositivity - Risk of genome integration due to viral replication in nucleus - Rapid clearance from serum depending on IgG, IgM titers and copper ions	- γ34.5 loci deletion	- Oncoselective replication	Markert et al. (2009); Stavrakaki et al. (2021); Fudaba and Wakimoto (2023)
							- decreased pathogenicity	
			T-VEC			- γ 34.5 loci deletion	- Oncoselective replication	Fukuhara et al. (2005); Hong et al. (2022); Kaufman et al. (2022); Shalhout et al. (2023)
						- ICP 47 deletion	- Decreased patho genicity	
						- GM-CSF expression inserted	- enhanced immune recruitment	
			G207			- γ 34.5 loci deletion	- Oncoselective replication	Markert et al. (2000); Markert et al. (2009); Foreman et al. (2017); Fudaba and Wakimoto (2023)
						- ICP6 inactivation	- Decreased pathogenicity	
							- Acyclovir sensitization	
			G47Δ			- γ 34.5 loci deletion	- Enhanced oncoselective replication	Fukuhara et al. (2005); Todo et al. (2022a); Todo et al. (2022b); Hong et al. (2022); Fudaba and Wakimoto (2023)
						- ICP6 inactivation	- Decreased pathogenicity	
						- α47 gene deletion	- Partial restoration of MHC I expression	
			rQNestin34.5v2			- Nestin-dependent ICP34.5 expression	- Enhanced oncoselective replication	Kambara et al. (2005); Chiocca et al. (2020)
	Poxviridae	Vaccinia Virus	VACV (wild-type)	- Large payload capacity (10–15 kb)	- Immunologically “cold” tumors may be resistant	N/A		Guo et al. (2019); Carpenter et al. (2021); Zhang et al. (2021); Zuo et al. (2021); Fudaba and Wakimoto (2023); Storozynsky et al. (2023)
				- Proven safety record in humans	- Radiotherapy-induced senescence limits virulence			
	Adenoviridae	Ad5	DNX2401/Δ24-RGD	- Negligible neurotoxicity - Mild AE profile - Production of anti-interferon proteins VA1 and VA2 results in resistance to immune clearance	- High prevalence of seropositivity - Reliance on Coxsackie-adenovirus Receptors for infectivity (relatively few on glial surface) - Lack of endogenous oncoselectivity- Ineffective neurotropism with systemic delivery without next-generation delivery techniques	- E1A deletion	- Selective replication in Rb-incompetent cells	Foreman et al. (2017); Lang et al. (2018); Cervera-Carrascon et al. (2019); Goradel et al. (2020); Gallego Perez-Larraya et al. (2022); van Putten et al. (2022); Zhu et al. (2022); Nassiri et al. (2023)
						- RGD incorporation	- Integrin-mediated cell entry	
			DNX2440/Δ24-RGDOX--			- E1A deletion	- Selective replication in Rb-incompetent cells	Foreman et al. (2017); Jiang et al. (2017); Cervera-Carrascon et al. (2019); Goradel et al. (2020); Fudaba and Wakimoto (2023)
						- RGD incorporation	- Integrin-mediated cell entry	
						- Addition of OX40L	- Enhanced tumor-specific T-cell activation	
			ONYX-015			- E1B deletion	- Selective replication in p53-incompetent cells (controversial)	Bischoff et al. (1996); Chiocca et al. (2004); Cervera-Carrascon et al. (2019); Goradel et al. (2020); Fudaba and Wakimoto (2023)
ssDNA	Parvoviridae	H-1PV	ParvOryx (wild-type)	- Nonpathogenic in humans	- Small size (low payload capacity)	N/A		Marchini et al. (2015); Carpenter et al. (2021); Fudaba and Wakimoto (2023)
				- Small size enables IV delivery				
+ssRNA	Picornaviridae	Poliovirus- Rhinovirus Chimera	PVS-RIPO	- Tropism to CD155 receptor (overexpressed on gliomas)	- Limited payload capacity	- Substitution of internal ribosome entry site with that of rhinovirus type 2	- Reduction of neurovirulence	Desjardins et al. (2018); Carpenter et al. (2021); Monie et al. (2021); Fudaba and Wakimoto (2023)
					- Significant AE profile			
	Flaviviridae	Zika Virus	ZIKV (wild-type)	- Endogenous neurotropism	- Risk of chronic neuroinflammation	N/A		Monie et al. (2021); Zhou et al. (2023)
				- Endogenous tropism to NSC and possibly GSC	- Poorly-understood teratogenic properties			
-ssRNA	Rhabdoviridae	Vesicular Stomatitis Virus	VSV (wild-type)	- Endogenous neurotropism	- Significant neurotoxicity	N/A		Cary et al. (2011); Monie et al. (2021)
				- Endogenous oncotropism	- Risk of chronic neuroinflammation			
				- High cytotoxic efficiency				
	Paramyxoviridae	Measles Virus	MV Edmonston’s	- Tropism to CD46 (overexpressed on certain glioma cells)	- Risk of subacute sclerosing panencephalitis	N/A		Allen et al. (2008); Monie et al. (2021); Fudaba and Wakimoto (2023)
			MV-CEA-			- addition of CEA	- enables noninvasive monitoring of viral titers	
		Newcastle Disease Virus	NDV-HUJ	- Robust immune activation	- unable to utilize tissue-specific promoter targeting	N/A		Zamarin and Palese (2012); Carpenter et al. (2021); Cuoco et al. (2021)
			MTH-68/H-	- Low seropositivity prevalence		N/A		
				- rarely pathogenic in humans				
				- lentogenic/mesogenic variants available				
dsRNA	Reoviridae	Mammalian Orthoreovirus	Reolysin™/Pelareorep/Type 3 Dearing (wild-type variant)	- Can be delivered IV - Rarely pathogenic in humans - Endogenouos oncoselectivity for Ras upregulation	- IV delivery has limited efficacy compared to IT	N/A		Foreman et al. (2017); Müller et al. (2020); Carpenter et al. (2021); Fudaba and Wakimoto (2023)
			(wild-type)			N/A		

### 2.2 Clinical translation of CNS oncolytic virotherapies

Impressive results such as patients surviving >11 years following repeat G47Δ administration indicate the vast potential of OV therapy in CNS cancer (Todo et al., 2022b). Accordingly, numerous trials are investigating CNS-targeted OVs, including for pediatric applications such as diffuse intrinsic pontine glioma (DIPG) and other midline tumors (Bernstock et al., 2023b; Fudaba and Wakimoto, 2023). Gallego Perez-Larraya et al. (2022) (NCT03178032) performed stereotactic intratumoral injection of the DNX-2401 oncolytic adenovirus in 11 pediatric patients with DIPG followed by radiotherapy; radiographically-evident remission was reported in 9 patients with partial response in a further 3. Notably, one patient survived over 38 months without tumor progression. Furthermore, immunohistochemistry of samples taken from autopsy depicted increases in CD8^+^ and CD4^+^ T-cells with a concomitant decrease in the immunosuppressive FoxP3^+^ regulatory T cells and M2 macrophages. Similarly, immunologic analysis of peripheral monocytes revealed increases in T-cell receptor clonality ascribed to enhanced production of extant T-cell clonotypes (Gallego Perez-Larraya et al., 2022). Other attempts at enhancing immunogenicity have utilized vectors tailored to induce dendritic cell recruitment. Umemura et al. (2023) reported results of a Phase I dose escalation trial (NCT01811992) utilizing adenovirus engineered to express FMS-like tyrosine kinase 3 ligand (Flt3L) and HSV1 Thymidine Kinase (HSV1-TK); these were delivered via intratumoral injection to patients with treatment-naïve high-grade glioma alongside valacyclovir and standard chemoradiation. Flt3L is a cytokine known to induce recruitment of dendritic cells and HSV1-TK expression enables *in situ* conversion of valacyclovir into a cytotoxic chemotherapeutic agent. The authors reported a median overall survival of 21.3 months, with 7 of 18 patients surviving for over 2 years. Furthermore, histopathological analysis of tumor recurrences demonstrated elevated populations of CD8^+^ T-cells and plasmacytoid dendritic cells (Bernstock et al., 2023c; Umemura et al., 2023). As such, attempts to mitigate obstacles to OV efficacy such as adequate delivery, immunogenicity, and fast immune clearance are underway (Carpenter et al., 2021).

While initial trials of OV candidates involved intravenous injection, current efforts are utilizing novel dosing and delivery approaches to maximize on-target transfection efficiency and parenchymal diffusion while minimizing the risk of systemic toxicity (Vogelbaum and Aghi, 2015). Intratumoral injection provides a means of circumventing the blood brain barrier. A phase II clinical trial in Japan resulted in the historic approval of G47Δ (UMIN000015995) which subsequently led to the commercial development of DELYTACT™ (Daiichi Sankyo, Tokyo, Japan) for glioma. Moreover, Todo et al. (2022a) demonstrated that repeated intratumoral dosing of OV candidates offers immune recruitment superior to that observed with a bolus dose, with significant increases in the amount of tumor-infiltrating lymphocytes associated with further OV dosing, and no statistically significant difference in adverse effect profile. Although not observed in the trial, repeated operative dosing (*i.e.*, stereotactic biopsy or injection) may carry a risk of surgical complications and pose an undue financial burden; ongoing efforts with advanced delivery techniques that facilitate repeated dosing may mitigate this (Bernstock et al., 2023b). Efforts utilizing viral packaging strategies have also been attempted. Fares et al. (2021) conducted a phase I clinical trial in which a conditionally-replicating adenovirus (CRAd-S-pk7) was packaged within neural stem cells and delivered via injection into the resection cavity with the intention of leveraging the inherent oncotropism of NSCs and their ability to migrate through the parenchyma. The authors reported that 83% of patients had stable disease and an overall survival of 18 months.

The oHSV G47Δ is a modification of an earlier oHSV known as G207. Todo *et al.* reported 84.2% survival at 1 year post G47Δ inoculation via intratumoral injection, sufficient for early termination, with over 25% of patients surviving more than 3 years after initial dose. Similarly, coadministration with immune modulators has been attempted as a means of improving OV efficacy. In particular, immunosuppressants such as cyclophosphamide and immune checkpoint inhibitors such as pembrolizumab (KEYTRUDA^®^, Merck, NJ, United States) may enable solutions to undesirably rapid viral clearance and anergic T-cell responses, respectively. A phase I clinical trial (NCT03152318) assessing the oncolytic herpesvirus rQNestin34.5v.2 with and without the alkylating agent and immunosuppressant cyclophosphamide has not yet reported results. However, Chiocca et al. (2020) reported promising signs of synergy between the OV candidate and cyclophosphamide in athymic mice bearing orthotopic xenografts of human U87ΔEGFR cell lines, with two mice treated with cyclophosphamide showing increased quantity of viral genetic material up to a month after inoculation. Nassiri et al. (2023) reported a phase II clinical trial (NCT02798406) in which 48 adult patients with recurrent GBM were treated with an initial injection of the oncolytic adenovirus DNX-2401 followed by infusions of pembrolizumab every 3 weeks. The authors observed a significant increase in overall survival with 52.7% of the 49-patient cohort alive at 12 months and further found 3 durable complete responses and a generally mild adverse event profile, justifying preparations for an eventual phase III trial (Zadeh et al., 2020; Nassiri et al., 2023). Such approaches are also being assessed in combination with enhanced delivery paradigms—another phase II clinical trial (NCT04479241) utilizing pembrolizumab as an adjuvant with convection-enhanced delivery-aided infusion of oncolytic poliovirus PVS-RIPO is ongoing and has yet to report results (Fudaba and Wakimoto, 2023).

## 3 Opportunities for SUMO-augmented oncolytic viral immunotherapies

While the regulatory approval of G47Δ in Japan marks a significant advancement in high-grade glioma treatment, challenges remain: despite initial responses patients frequently progress. While the results from these clinical trials are certainly encouraging, this trend suggests that significant space for further benefit yet remains. As such, it is increasingly evident that combination approaches, both with conventional treatment regimens and newer adjuvants, are likely required to address challenges posed by the CNS environment. Several nodes within the SUMO pathway could serve as targets for adjunct therapies to increase OV efficacy by enhancing viral replication and persistence and modulating the type I interferon response. The SUMO pathway, for example, has numerous interactions with both the innate and adaptive immune systems (Adorisio et al., 2017). Furthermore, some viruses utilize the SUMO pathway as a means of coopting cellular processes or subverting the immune response (Fan et al., 2022). To this end, SUMOtherapeutics may serve to combine the advantages of individual platforms to overcome the clinical challenges posed by high-grade glioma.

Compounds such as histone deacetylase inhibitors (HDACi) have been observed to impact the SUMO pathway and enhance OV efficacy. Otsuki *et al.* assessed the ability of valproic acid, an antiepileptic agent with known histone-deacetylase inhibitory activity and pro-SUMOylation characteristics, to enhance *in vitro* and *in vivo* oHSV infectivity (Otsuki et al., 2008; Sang et al., 2016). Inoculating human glioma cell cultures with a GFP-tagged oHSV-1 mutant after pre-treatment with valproic acid resulted in significant (>100-fold) increases in viral expression in U251 human glioma cells. Furthermore, significant decreases were reported in interferon-responsive gene products such as STAT1, PKR, and PML in glioma cultures inoculated with oHSV after valproic acid pretreatment, suggesting that benefits were attributable to immunomodulatory properties of valproic acid. Finally, the authors observed a significant survival benefit in nude mice bearing orthotopic U87ΔEGFR tumors that were pretreated with valproic acid prior to viral inoculation, with a 60-day survival of 50% compared to 20% in mice treated with virus alone and 0% for control (Otsuki et al., 2008). Similarly, Kawamura et al. (2022) observed significant increases in intratumoral replication of oHSV in *in vivo* malignant meningioma models following treatment with trichinostatin A and panabinostat, other HDACi with known pro-SUMO activity. This synergy has also been observed in other OV candidates such as vesicular stomatitis virus and human adenovirus 5 recombinants (Nguyen et al., 2010). However, to the best of the authors’ knowledge, the interactions between HDACi and SUMO have yet to be comprehensively described in the context of viral infection.

Even though the potential benefit offered by SUMOtherapies is significant, it is important to note challenges yet to be overcome as well as areas for further development. Regarding the former, the challenges associated with drug delivery both to the tumor and to disseminated disease within the brain as well as the notoriously challenging tumoral heterogeneity of CNS malignancies remain to be addressed. As to the latter, while no biomarkers predictive of response have been reported at time of writing, further research into the role of SUMO and other post-translational modifications on OV efficacy may lend insights into response for combination therapies. Markers such as Myc, CDK6, and Cyclin D1 may be potential candidates given previous reports of their interactions with both SUMO and glioblastoma pathogenesis, however studies assessing biomarkers in combination with SUMOtherapeutics and OV therapies have not yet been conducted (Kessler et al., 2012; Stavrakaki et al., 2021).

### 3.1 Enhancing viral replication and persistence

A plethora of viruses have been observed to directly inhibit the SUMO pathway components related to immune/inflammatory responses and/or subvert SUMO machinery to enhance replication. In the case of HSV-1, preclinical research has reported a role for promyelocytic leukemia nuclear bodies (PML-NBs), themselves regulated by SUMOylation (Imbert et al., 2022), in mitigating HSV-1 infectivity. PML-NBs are protein complex products of interferon-stimulated genes and thought to be a component of the antiviral response. For example, work by Liang et al. (2016) found that SUMOylation at the lysine 160 residue of PML is necessary for recruitment of PML-NB components. The authors also observed that SUMO2/3ylation of PML resulted in disruption of formed PML-NBs.

SUMO pathway components also play a role in responses to HSV-1 infection. After HSV-1 infection, PIAS1 has been found to traffic to the nucleus and participate in restriction of HSV-1 genome transcription in a manner complementary to PML. In response, a viral E3 ubiquitin ligase known as ICP0 counteracts PIAS1 in a non-destructive manner as a means of alleviating this restriction. Similarly, another HSV-1 viral protein known as ICP27 was observed by Kim et al. (2017) to repress NF-κB activity by inhibiting the SUMOylation of Daxx, an endogenous anti-inflammatory protein. It follows that greater awareness of the interplay between SUMO and the HSV-1 proteome may yield a variety of targets for SUMOtherapeutics identified with modern high-throughput screening methods.

Human adenoviral proteins interact with host cell components in a manner similar to that observed with HSV-1; Endter et al. (2001) identified a SUMO-interaction motif in the adenoviral oncoprotein E1B-55K and demonstrated via knockout experiments in rats that a SUMO-E1B-55K interaction is necessary for the nuclear localization of adenoviral proteins. Muller and Dobner (2008) subsequently reported that the same protein upregulates the SUMOylation of p53, enabling greater transformation efficacy. More recent work by Muncheberg et al. (2018) reported that SUMOylated E1B-55K causes the RNF4-dependent ubiquitinylation and degradation of Daxx, demonstrating that RNAi-induced knockout of E1B-55K caused significant reductions in adenoviral gene expression in infected cells. Similar to the role of the herpes ICP0 protein as an E3 ligase, the adenoviral E4-ORF3 protein was found by Sohn and Hearing (2016) to function as both an E3 ligase as well as a SUMO-polymerizing elongase. Finally, Higginbotham and O'Shea (2015) implicated both E1B-55K and E4-ORF3 in the recruitment of SUMO2/3ylated E2A viral genomic replication domains, imputing SUMOylation in the evasion of intracellular antiviral activity to facilitate viral replication.

While HSV-1 and Ad5 recombinant viruses represent the leading edge of CNS oncolytic virotherapy, SUMO has also been implicated in similarly critical roles within other OV candidates that have reached clinical testing. These include poliovirus (Pampin et al., 2006), vaccinia virus, and reovirus (Yu et al., 2016). Accordingly, selective modulation of the SUMO pathway may present several novel adjuvant therapeutic targets (Carpentier and Meng, 2006; Woroniecka et al., 2018).

### 3.2 Effects on T Cell populations

In the OV context, efficacy via the immunostimulatory mechanism is predicated on effective T-cell mediated responses for both oncolysis and development of antitumoral immunity (Chiocca and Rabkin, 2014). Perhaps unsurprisingly, SUMO serves as a high-level modulator of several processes critical for both immunogenic and immunosuppressive T-cell responses as well as the tumor escape mechanisms affecting them (Sajeev et al., 2021). As such, modulation of SUMO components may serve as a means of augmenting OV therapies by modulating the function of key T-cell populations.

Regulatory T-cells (Treg) are a CD25^+^ T-cell subclass that suppress immune responses via secretion of TGF-β2 and IL-10 and shift of the cytokine profile towards the Th2 type. The role played by Treg cells is critical: Fecci *et al.* demonstrated not only a correlation between Treg fraction and CD4^+^ T-cell proliferative defects but also that depletion of Treg populations can induce spontaneous rejection of murine malignant astrocytomas in a VM/Dk mouse model (Fecci et al., 2006). As such, the reliance of Treg expansion and function on SUMO serves as an appealing target. Ding *et al.* reported that the knockout of UBC9 (the SUMO E2 ligase) in a Treg population resulted in impaired proliferation, activation, and suppressive functionality (Weitz et al., 2022). Similarly, Lam et al. (2023) treated T-cell populations from patients with chronic lymphocytic leukemia with TAK-981 and reported decreased Treg differentiation. As such, targeted inhibition of Treg activity may be feasible with SUMOtherapeutics.

The SUMO pathway is also implicated in the functioning of non-inhibitory effector T cells required for the development of antitumoral immunity. Lam et al. (2023) reported enhanced secretion of IFNγ by CD4^+^ and CD8^+^ populations alongside enhanced T-cell-mediated cytotoxicity in OCI-LY3 lymphoma cultures after treatment with TAK-981. Other preclinical work has identified the specific role of various SUMO components in effector T-cell homeostasis and adaptations to the tumor microenvironment. Wu et al. (2022) observed that SENP7 served as a critical oxidative stress sensor in CD8^+^ T-cells, mediating deSUMOylation of PTEN and enhancing antitumor function while maintaining metabolic state in the face of the tumor microenvironment. Further inquiry aimed at elucidating the specific nature of SUMO- effector T-cell interactions may provide novel insights and yield potential therapeutic candidates.

Finally, SUMOylation has also been reported to be a key mediator of immune escape strategies that attenuate both innate and OV-instigated antitumoral T-cell efficacy. Programmed death-ligand-1 (PD-L1) is an inhibitory molecule that suppresses the antitumoral functions of effector T-cells and is the target of many modern immunotherapeutic approaches. Bernstock et al. (2017b); Bernstock J. et al. (2017c) demonstrated that topotecan, a topoisomerase I inhibitor with known SUMO-inhibition properties, suppresses PD-L1 expression, reporting a nearly 4-fold reduction in PD-L1 expressed by LN229 cultures upon treatment with 10 µM topotecan. Trogocytosis, the process of transferring cell membrane fragments between cells in contact, has been posited to be another such mechanism of tumor immune escape. Lu et al. (2022) treated MC38-OVA murine colonic adenocarcinoma cultures with TAK-981, reporting decreased trogocytosis and thus preserved viability and functionality of cytotoxic T lymphocytes. Another mechanism of escape results in inhibition of MHC-I expression, impeding the ability for T-cells to identify and destroy cancer cells. Demel et al. (2022) demonstrated that hyperSUMOylation in diffuse large B-cell lymphoma cultures contributed to suppression of MHC I antigen presentation machinery and thus neutralization of T-cell efficacy. The authors treated DLBCL cultures with TAK-981 and observed 2-fold increases in MHC-I expression as well as IFNγ-induced STAT1 phosphorylation. As such, the importance of SUMO in both physiologic and pathologic T-cell mechanisms makes it a promising target to enhance innate antitumoral activity as well as OV efficacy.

### 3.3 Modulating the type I interferon response

The Type I Interferon response to viral invasion is of clinical interest. Comprised of 13 IFNα subtypes, IFNβ, and various poorly delineated single gene products, the Type I IFN response plays a crucial role in modulating the host response against a variety of pathogens including viruses (Adorisio et al., 2017). The Type I IFN response can be a double-edged sword—with an excessive response resulting in autoimmune damage of healthy tissue/clearance of viral vectors, while a meagre response can blunt immune sensitization-based therapeutic approaches (McNab et al., 2015).

**FIGURE 3:**
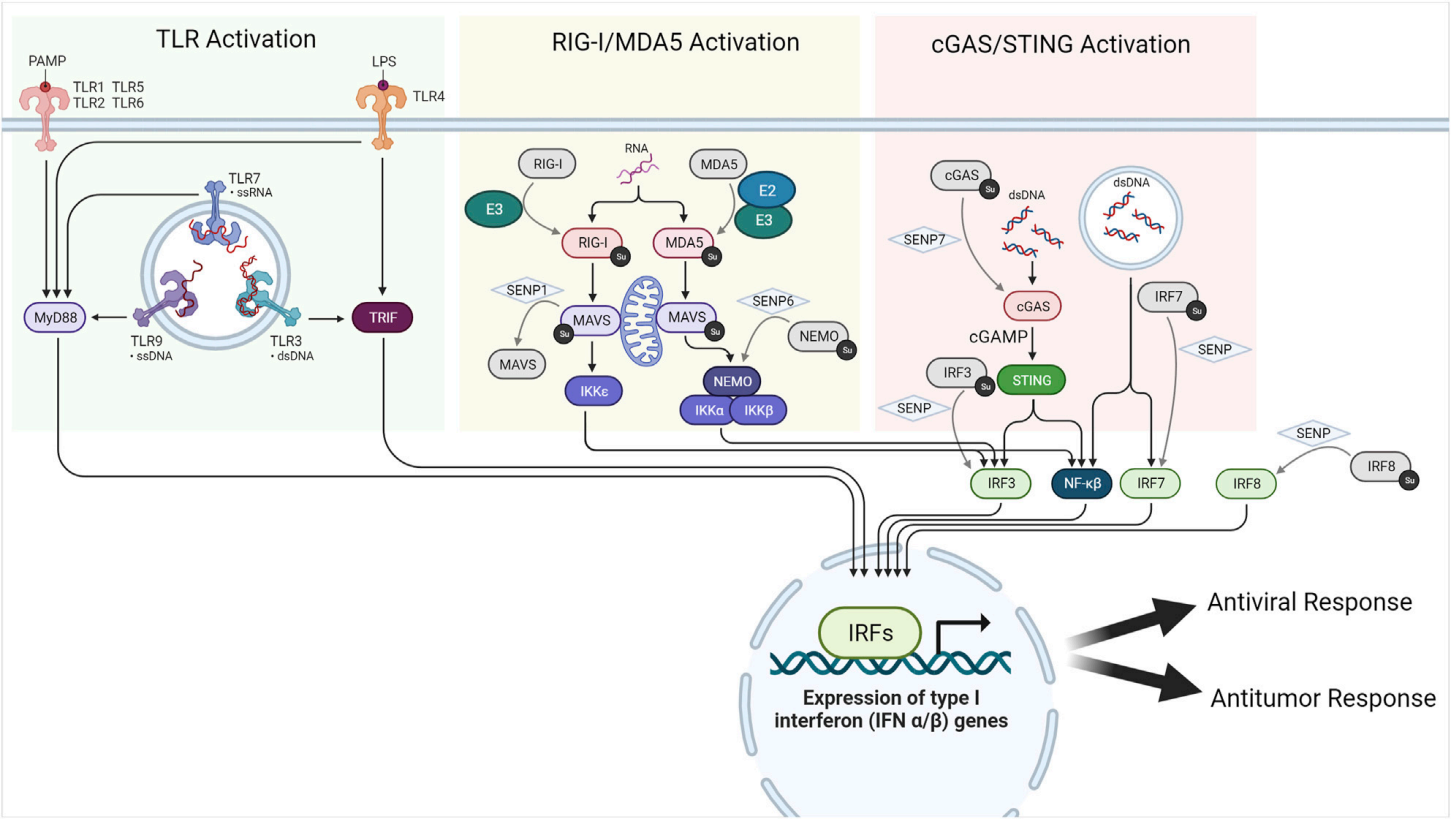
SUMO and the type I interferon response.

There are four primary methods by which the IFN response is triggered, all of which utilize Interferon Regulatory Factor (IRF) 3 or IRF7 as regulators. They include: a) detection of abnormal intracellular DNA via cGMP-AMP Synthase (cGAS); b) detection of abnormal intracellular RNA via RIG I-like Receptors (RLRs); c) TRIF-mediated detection of PAMPs via TLR 3 and 4; and d) PAMP detection via TLR 7 and 9 (Crowl and Stetson, 2018). Knockout studies have suggested a role for SUMO in both upregulation and downregulation of the Type I IFN response (Figure 3). Crowl and Stetson observed a IRF3/IRF7-independent downregulation of the Type I IFN response in wild-type murine cells when compared with SUMO2/3 knockout murine cultures (Crowl and Stetson, 2018). Moreover, Kubota et al. (2008) reported that SUMOylation of IRF3 and IRF7 helped attenuate the Type I IFN response evoked when murine cell cultures were inoculated with vesicular stomatitis virus. Work by Chang et al. (2012) also imputed SUMO2/3ylation of IRF8 as an inhibitor of Interferon-related gene production in resting macrophages, additionally demonstrating the intrinsic role of SENP1 in deSUMOylating IRF8 upon the activation of macrophages and potentiation of the immune response. Finally, Liu et al. (2013) reported that SENP6 played a critical role in the deSUMO2/3ylation of NEMO and prevention of NF-κB-induced inflammation, observing significant increases in TNF-α, IL-6, and 30-h mortality in mice depleted of SENP6 via siRNA and challenged with LPS when compared to control. While much of the literature discusses the immunosuppressive role of SUMO, it is important to note that effective propagation of the Type I IFN response is dependent on SUMOylated substrates.

Although the role of SUMO is being increasingly detailed in preclinical literature, only one SUMOtherapeutic directly targeting the Type I Interferon response, TAK-981, is presently under clinical investigation (NCT03648372, NCT04074330, NCT04776018, NCT04381650). TAK-981 is a small-molecule irreversible inhibitor of the E1 ligase that functions by preventing transfer of SUMO1 or SUMO2/3 to E2 ligase (also known as Ubc9) (Figure 1). Lightcap et al. (2021) observed that inhibition of SUMOylation via this compound resulted in increased phosphorylation of STAT1 and STAT2 in human B-cell lymphoma cultures and increased expression of IFNβ and other IFN-stimulated genes in mouse splenocytes and human T-cell lines. The authors observed similar upregulation in IFNβ and other ISG products *in vivo* when BALB/c mice bearing subcutaneous A20 murine B-cell lymphomas were administered intratumoral microdoses of TAK-981. To assess the ability of TAK-981 to potentiate protective antitumoral responses, C57BL/6 mice were further exposed with a combination of ovalbumin and TAK-981 prior to implantation of B16F10-OVA murine melanoma tumors and reported statistically significant increases in IFNγ and Granzyme B in mice treated with TAK-981 and vehicle. Moreover, the authors also reported reductions in tumor volume in mice exposed to TAK-981 and ovalbumin at 30 days post tumor implantation, similar to tumor reductions in mice exposed to a known TLR3 agonist and ovalbumin. As such, these reports provide promising *in vivo* and *in vitro* evidence that SUMO is a viable target for modulating the antitumoral immune response (Lightcap et al., 2021). While TAK-981 is currently in clinical trials as an immunosensitizing agent for advanced solid tumors with and without co-administered immune checkpoint inhibitors, the potential as an adjuvant for OV therapy makes it of further research interest (Zhu et al., 2022).

SUMO is deeply involved in regulating the Type I interferon response generated by detection of non-self DNA and RNA. The intracellular RNA sensors RIG-I and MDA5 and the associated MAVS enzymes require SUMOylation to induce expression of ISGs. On the other hand, deSUMOylation of the DNA sensor cGAS and key regulatory factors such as IRF3, IRF7, and IRF8 is required for ISG expression.

#### 3.3.1 cGAS/STING

The Stimulator of Interferon Genes (STING) and GMP-AMP synthase (cGAS) are proteins that serve as integrators of various pattern recognition receptors (PRRs) to detect non-self molecules and stimulate host defense (Reinert et al., 2016). If these PRRs encounter a non-self molecular pattern within the intracellular milieu, STING undergoes a conformational change, in turn activating the Type I Interferon pathway and production of pro-inflammatory cytokines via interactions with IRF3 and NF-κβ. Next, cGAS serves as a cytosolic DNA sensor that produces cyclic GMP-AMP (cGAMP), a potent activator of STING; cGAMP is also able to translocate to adjacent uninfected cells and potentiate anti-viral responses (Lee et al., 2019). Similarly, for OV, Bommareddy et al. (2019) found that STING expression attenuates the oncolytic properties of the oHSV T-VEC in an *in vivo* melanoma culture. Conversely, STING participates in anti-cancer surveillance and serves to activate the immune system against the nascent tumor (Lee et al., 2019). In fact, Haase et al. (2022) demonstrated that the downregulation of DNA repair pathways in H3.3-G34R/V type high-grade gliomas enabled greater intrinsic STING-mediated anti-tumoral immunity and amplified the therapeutic efficacy of chemoradiation in mouse models. As such, the repressed STING expression secondary to hypermethylation of the STING promoter observed in many high grade primary brain tumors may offer an explanation for their resistance to treatment (Low et al., 2022; Qiu et al., 2022). While the combination of cGAS and STING has been shown to be a potent defense against infection with HSV-1 and a potential obstacle to HSV-based OVs, the nuanced role of STING in generating both desirable and undesirable immune responses necessitates measures to preserve STING function (Ran et al., 2011).

SUMO is critical for the persistence and function of the cGAS/STING pathway (Yu et al., 2022). Hu et al. (2016) reported the role of TRIM38-mediated SUMOylation of cGAS and STING in preventing their ubiquitination and degradation. Conversely, Cui et al. (2017) found that deSUMOylation of cGAS by SENP7 enhances DNA-binding ability and thus immune activation. The authors also demonstrated that SENP7 knockout mice were significantly more susceptible to infection with HSV-1, with 100% mortality at 3 days (20% in control mice) and 15-fold reductions in IFNβ as measured by ELISA (Cui et al., 2017). These modifications are thought to be a means of maintaining a dynamic reserve of DNA sensors for rapid response to pathogenic insult while simultaneously preventing inappropriate or spontaneous activation (Yu et al., 2022).

#### 3.3.2 RIG-1/MDA5

RIG-1 (retinoic acid-inducible gene 1) and MDA5 (melanoma differentiation-associated gene 5) are both members of a group of intracellular PRRs collectively known as RIG-I-like receptors. These proteins serve as intracellular RNA sensors for both pathogenic exogenous and aberrant endogenous RNA; these are, therefore, important defenses against infection and malignant transformation (Jiang et al., 2023). This makes modulation of the RIG-1/MDA5 response of interest for both OVs and anti-tumor immunotherapies, particularly those based in immune-sensitization and checkpoint blockade. Preclinical investigation of such approaches is already underway: Marek et al. (2023) demonstrated a synergistic cytotoxic effect upon utilizing a vesicular stomatitis virus-Newcastle disease virus chimera as a means of stimulating RIG-1 in concert with anti-CTLA-4-based checkpoint inhibitor therapy.

In light of this, the SUMO pathway’s involvement in enhancing the pro-inflammatory effect of RIG-1 and MDA5 (Jiang et al., 2023). SUMOylation of RIG-1 and MDA5 via TRIM38 (a SUMO E3 ligase) inhibits their ubiquitin-mediated degradation and dephosphorylation via protein phosphatase 1 (Hu et al., 2017). Similarly, the SUMO E2 and PIAS2β (another SUMO E3 ligase) are involved in SUMOylation and activation of MDA5 (Fu et al., 2011). Furthermore, the mitochondrial antiviral signaling protein (MAVS) requires SUMOylation to participate in the response to RIG-1 activation (Dai et al., 2023). Accordingly, development of compounds targeted at individual components of the SUMO pathway interfacing with the RIG-1/MDA5 component of the Type I interferon response may provide novel/selective methods of modulation of the immune response against OV candidates.

### 3.4 Augmenting other immunotherapeutic approaches

The function of the SUMO pathway as a means of modulating the immune response makes it a potentially potent method of augmenting other immunotherapeutic treatment strategies (Chen, 2023). Methods such as CAR-T cells and cancer vaccines that have represented advances in the management of other cancers are thought to have faltered against glial malignancies due to the “cold” immunologic microenvironment and relative dearth of neoepitopes for activation of anti-tumoral immunity (Bagley et al., 2018; Maggs et al., 2021). Although SUMO is known to play a role in the virulence of glioma, a greater awareness of the SUMO proteome and its interface with glial tumor immune privilege may yield novel therapeutic targets—SUMO-pathway targeting is already being assessed clinically as a therapeutic modality for a variety of solid tumors using TAK-981. Notably, one of the aforementioned clinical trials is also investigating TAK-981 with the immune checkpoint inhibitor pembrolizumab as an adjuvant, similar to some of the ongoing trials for OVs. As such, the fact that the SUMO pathway governs both cellular replication and cycling as well as immune responses makes it a shared node between two otherwise orthogonal approaches—identifying therapeutic targets within this space has the potential to improve the synergy between combination treatments and minimize the chance of resistance development (Kroonen and Vertegaal, 2021).

Even so, the lack of a comprehensive understanding of the role of SUMO within healthy cells and malignant cells, poses a challenge as targeting nodes within a pathway as broad-reaching as SUMO may have unforeseen downstream implications. However, the development of modern proteomics technologies and high-throughput screening systems has brought the attainment of such an understanding within reach (Bernstock et al., 2016; Bernstock et al., 2018). Future cancer therapies designed to take advantage of such an understanding may overcome the hurdles facing effective OV deployment for CNS malignancies.

## 4 Conclusion

Modulation of the SUMO pathway as an adjunct to OVs may enhance the replication and persistence of OVs, dampen resulting overactive immune responses, augment the development of apropos anti-tumoral immunity, and/or enable greater synergy with other immunotherapies; these strategies may help replicate OV successes in the CNS. Furthermore, as ongoing clinical trials assess means to overcome obstacles to OV efficacy as well as the feasibility and safety of combination therapies, directed preclinical inquiry into the role of SUMOtherapeutics as an adjuvant for OVs is required. In concert with such preclinical efforts, ongoing OV trials and future clinical investigation of OV-SUMOtherapy-Immunotherapy combinations may produce valuable additional treatment options for high-grade gliomas.
